# *Alcohol dehydrogenase, iron containing, 1* promoter hypermethylation associated with colorectal cancer differentiation

**DOI:** 10.1186/1471-2407-13-142

**Published:** 2013-03-22

**Authors:** Chung Hyun Tae, Kyung Ju Ryu, Seok-Hyung Kim, Hee Cheol Kim, Ho-Kyung Chun, Byung-Hoon Min, Dong Kyung Chang, Poong-Lyul Rhee, Jae J Kim, Jong Chul Rhee, Young-Ho Kim

**Affiliations:** 1 Department of Medicine, Samsung Medical Center, Sungkyunkwan University School of Medicine, Seoul, Republic of Korea; 2 Samsung Biomedical Research Institute, Samsung Medical Center, Seoul, Republic of Korea; 3 Department of Pathology, Samsung Medical Center, Sungkyunkwan University School of Medicine, Seoul, Republic of Korea; 4 Department of Surgery, Samsung Medical Center, Sungkyunkwan University School of Medicine, Seoul, Republic of Korea

**Keywords:** ADHFE1, Promoter methylation, Colorectal cancer, Differentiation

## Abstract

**Background:**

The aberrant methylation of CpG islands in the promoter is associated with colorectal cancer (CRC) carcinogenesis. In our previous study, the promoter of alcohol dehydrogenase, iron containing, 1 (*ADHFE1*) was most highly methylated in CRC compared to normal colorectal mucosa. In this study, we examined the expression and function of the *ADHFE1* in CRC.

**Methods:**

We examined the promoter methylation and mRNA expression of *ADHFE1* with 5-aza-2^′^-deoxycytidine (5-Aza-2-dC) in 12 CRC cell lines, 124 paired CRC and adjacent normal mucosa, and 59 advanced adenomas. To confirm methylation of *ADHFE1*, we performed bisulfite genomic sequencing in 3 CRC cell lines, 6 paired CRC and adjacent normal mucosa. ADHFE1 protein expression was studied using western blot and immunohistochemistry, respectively in the 36 and 243 paired CRC and adjacent normal tissue. We transfected the DLD-1 with pcDNA3.1 vector containing *ADHFE1* and examined the expression of differentiation marker, such as ALP, CEA and Cdx2. We examined the ADHFE1 expression at distinct developmental stages in mouse embryos.

**Results:**

The *ADHFE1* promoter was hypermethylated in all CRC cell lines, 81.8% in CRCs, and 84.7% in advanced adenomas, with reciprocal change by 5-Aza-2-dC. The expression of *ADHFE1* mRNA was down-regulated in all CRC cell lines and 96.3% in CRC tissues. The expression of ADHFE1 protein was down-regulated in 91.7% of CRC tissues. In the immunohistochemistry, normal epithelial cells at the crypt top showed very strong ADHFE1 expression, whereas they were much weaker at the crypt base. In CRC, the good differentiation was significantly associated with high ADHFE1 expression. The activity of differentiation marker, such as ALP and CEA, was higher in pcDNA3.1-*ADHFE1* transfected CRC cells with consistent correlation with ADHFE1 protein than control. In mouse embryos, ADHFE1 in the large intestine was the first detected at E15.5. At E18.5, ADHFE1 was predominantly expressed in the top of the mature crypt epithelium.

**Conclusions:**

It showed that the hypermethylation of *ADHFE1* promoter in CRC is concordance with down-regulation of *ADHFE1* mRNA and ADHFE1 protein. ADHFE1 has an important role of differentiation in CRC, as well as normal colorectal mucosa and embryonic developmental processes.

## Background

Colorectal cancer (CRC) is one of the leading causes of cancer-related death in the world, in spite of the availability of colonoscopy screening and effective prevention through a colorectal adenoma resection. Recent evidence indicates that epigenetic alterations, apart from genetic alteration, play an important mechanism in CRC carcinogenesis [1]. Epigenetic alterations are potentially reversible in neoplasia, and they present new opportunities for the clinical management of cancer, which are different from mutation [2]. The most common epigenetic alteration in CRC is aberrant DNA methylation, in which a methyl group is added to the cytosine base in the dinucleotide sequence CpG islands, which are often associated with the promoter [1,2]. Methylation in a gene promoter region generally correlates with a silenced gene [2]. Several genes involved in CRC carcinogenesis found to be silenced by DNA methylation include: *MCC, MLH1, MGMT, APC, hMLH1, GATA-4, GATA-5, TFPI2,* and *SOX17*[2-8].

The iron-activated alcohol dehydrogenase family had seemed to only exist in microbial organisms until Mao *et al.* cloned alcohol dehydrogenase, iron containing, 1 (*ADHFE1*) from a human fetal brain cDNA in 2002 [9]. About 10 years later, just as there are only a few more studies [10,11]. Kim *et al.* showed that *ADHFE1* transcript exhibits differentiation-dependent expression during *in vivo* brown and white adipogenesis [10]. Another study indicated that *ADHFE1* is related to bacterial γ-hydroxybutyrate dehydrogenase and has a conserved NAD-binding site [11]. Recently, we had documented gene profiles with promoter hypermethylation using Human Methylation27 DNA Analysis BeadChip in CRC [12]. Among these genes, the *ADHFE1* gene is the most highly methylated in CRC compared to normal colorectal mucosa and the expression is down-regulated more than twofold [12]. It was also reported that the *ADHFE1* promoter is hypermethylated in CRC and adenoma by another research group [13].

Here, we investigated *ADHFE1* promoter methylation in CRC cell lines and CRC tissues. Additionally, we researched the functional implications of *ADHFE1* methylation for CRC.

## Methods

### Subjects

One hundred twenty four CRC and 59 advanced adenoma tissues greater than 1 cm in diameter and/or with a villous component and/or with severe dysplasia were collected from patients treated at Samsung Medical Center, Seoul, Korea (Table 1). We also retrieved 124 adjacent normal colorectal mucosa tissues from CRC patients. None of the patients had clinically apparent polyposis syndrome or hereditary nonpolyposis colon cancer syndrome. Written informed consent was obtained from all patients. This study was reviewed and approved by the Samsung Medical Center Institutional Review Board in accordance with the Declaration of Helsinki.

**Table 1:** Clinical characteristics of the study population

**CRC (n = 124)**	
Age, years	
Mean ± SD	58.9 ± 9.97
Median (range)	60.0 (32–77)
Gender (%)	
Male/Female	77 (62.1)/47 (37.9)
Location (%)	
Right/Left side	64 (51.6)/60 (48.4)
Differentiation (%)	
Well/Moderate/Poorly	13 (10.5)/92 (74.2)/19 (15.3)
Duke stage (%)	
I/II/III/IV	4 (3.2)/64 (51.6)/45 (36.3)/11 (8.9)
Microsatellite instability status (%)	
MSS/Low MSI/High MSI	107 (86.2)/8 (6.5)/9 (7.3)
**Advanced adenoma (n = 59)**	
Age, years	
Mean ± SD	46.5 ± 1.74
Median (range)	40.0 (17–72)
Gender (%)	
Male/Female	47 (79.7)/12 (20.3)
Size, mm	
Mean ± SD	17.4 ± 0.92
Median (range)	15.0 (0.7-35)
Location (%)	
Right/Left side	20 (33.9)/39 (66.1)
Histology (%)	
Tubular/Tubulovillous/Villous	39 (66.1)/19 (32.2)/1 (1.7)
Grade of dysplasia (%)	
Low/High	49 (83.1)/10 (16.9)

CRC, colorectal cancer; SD, standard deviation; MSS, microsatellite stability; MSI, microsatellite instability.

### CRC cell lines and 5-aza-2^′^-deoxycytidine (5-aza-2-dC) treatment

We used CoLo205, DLD-1, HT15, HT116, HT29, KM12C, KM12SM, KM20, RKO, SNU81, SW48 and WiDr as human CRC cell lines. Each of the CRC cell lines were treated for 48 hours with 0 or 2 μM 5-aza-2-dC (Sigma-Aldrich, St. Louis, Mo, USA).

### DNA extraction and bisulfite modification

Genomic DNA was extracted using the QIAamp tissue kit (Qiagen, Valencia, CA, USA). A bisulfite conversion was performed using the Zymo EZ DNA methylation kit (Zymo Research, Irvine, CA, USA) according to the manufacturer’s protocol. DNA was extracted from tumor cells or the epithelial layer of formalin-fixed, paraffin embedded tissue sections.

### Quantitative real time PCR (RT-PCR) to measure DNA methylation (MethyLight)

The *ADHFE1* gene presents on human chromosome 8q 13.1. The promoter region of the *ADHFE1* gene contains a dense CpG island located from nucleotides −257 to +265 relative to translation start site (TSS). We examined 12 CRC cell lines and CRC tissues using methylation-specific PCR with primers located from −4 to +102 relative to TSS.

After bisulfite conversion of the same amount of DNA solution, *ALU*-based MethyLight control reaction was done to quantify the number of input target DNA, and the threshold cycle values were confirmed to be comparable among DNA samples. The primer and probe sequences used were as follows: *ADHFE1* forward primer: 5-CGTTATGGTCGTTGTCGTTC-3^′^, *ADHFE1* probe: 6FAM-CGCCGACCCCGCACTCACGC-MGBNFQ, *ADHFE1* reverse primer: 5-GTAAACACCCTACGATCCCCTACCCG-3. *ALU* forward primer: 5-GGTTAGGTATAGTGGTTTATATTTGTAATTTTAGTA-3, *ALU* probe: 6FAM-CCTACCTTAACCTCCC-MGBNFQ, *ALU* reverse primer: 5-ATTAACTAAACTAATCTTAAACTCCTAACCTCA-3.

MethyLight was performed by using a 7900HT Real-Time PCR system (Applied Biosystems, Foster City, CA, USA). The PCR program was as follows: 5 minutes at 95°C, followed by 45 cycles of 30 seconds at 95°C, 30 seconds at 60°C, and 30 seconds at 72°C. CpGenome™ universal methylated and unmethylated DNA (Chemicon, Temecula, CA, USA) were used as a positive control for the methylated and unmethylated genes, respectively. The percentage of methylated reference (PMR) at a specific gene was calculated by dividing the *GENE*/*ALU* ratio of a sample by the *GENE*/*ALU* ratio of the *in vitro* fully methylated placental DNA and multiplying by 100. We used the PMR cut-off value more than 10% to define methylation-positive *vs.* negative and determined DNA methylation frequencies for each CpG island locus.

### Bisulfite genomic sequencing

We performed bisulfite genomic sequencing for CRC tissue, and adjacent normal colorectal mucosa according to the manufacturer’s instructions. We performed PCR to amplify the sequence for CpG island located in the promoter lesion and TSS using the following primer sets: BS1F (5^′^-TTTGAAAAATAAGATATAGTGTTTAATTAT-3^′^) and BS1R (5^′^-AAATAACTCTAAACTCAACAAAAAC-3^′^); BS2F (5^′^-GTTTTTGTTGAGTTTAGAGTTATTT-3^′^) and BS2R (5^′^-ACCACTACCCCAAAATTACAC-3^′^). The PCR assays were performed at 95°C for 15 minutes, 45 cycles of 92°C for 30 seconds, 50 (BS1) or 57 (BS2)°C for 45 seconds, 72°C for 30 seconds, followed by a final extension at 72°C for 10 minutes. PCR products were cloned using One Shot® TOP10 kit (Invitrogen, Carlsbad, CA, USA) and sequenced using 3730xl DNA analyzer (Applied Biosystems, Foster City, CA, USA).

### RT-PCR

Two μg of total RNA was reverse transcribed by M-MuLV reverse transcriptase with Oligo(dT)16 as a primer. RT-PCR was performed on the ABI PRISM 7900 sequence detection system (Applied Biosystems, Foster City, CA, USA) by monitoring the increase of fluorescence by the binding of SYBR Green (TaKaRa, Otsu, Japan) to double-stranded DNA. A dissociation analysis was performed at the end of each PCR reaction to ensure there was only a specific product. The settings for the PCR thermal profile were as follows; initial denaturation at 95°C for 5 minutes, followed by 45 amplification cycles of 95°C for 10 seconds, annealing at 55°C for 10 seconds, and elongation at 72°C for 30 seconds. The primer and probe sequences used were as follows: *ADHFE1* forward primer: 5- CACTGCCAGGATCCAAGATG −3^′^, *ADHFE1* reverse primer: 5- GAGCTTTGGGGAATTTCCTG −3. *GAPDH* forward primer: 5^′^ - CCACCCATGGCAAATTCCATGGCA - 3^′^, *GAPDH* reverse primer: 5^′^ - TCTAGACGGCAGGTCAGGTCCACC -3^′^. Each PCR was run in triplicate. For quantification of gene expression changes, the ΔΔCt method was used to calculate relative fold changes normalized against the *GAPDH* gene.

### Western blot

Cells lysates were prepared in ice-cold RIPA buffer (0.5% sodium deoxycholate, 1% Nonidet P-40, 150 mM NaCl, 50 mM Tris (pH 7.5), 0.1% SDS, and 1 mM PMSF) and cleared by microcentrifugation (14 000 rpm for 30 min at 4°C). The protein concentration in each sample was estimated by BCA Assay. Twenty to thirty μg of each protein sample was resolved by 12% SDS–polyacrylamide gel electrophoresis, and electroblotted into nitrocellulose membranes. After 1 hour incubation in blocking solution (5% non-fat milk in TBST), it was incubated with primary antibodies overnight at 4°C. The primary antibodies included: those against ADHFE1 (1:1000) (Sigma-Aldrich, St. Louis, MO, USA), carcinoembryonic antigen (CEA) (1:1000), caudal type homeobox 2 (Cdx2) (1:1000) (Cell Signaling, Danvers, MA, USA) and β-actin (1:5000) (Santa Cruz Biotchology, Santa. Cruz, CA, USA). The blots were then washed in TBST and incubated with HRP-conjugated secondary antibodies for 1 hour at room temperature. The results were visualized using an ECL system (Amersham Pharmacia Biotech, Arlington Heights, IL, USA).

### Immunohistochemistry

To confirm the ADHFE1 protein expression in CRC and adjacent normal colorectal mucosa, we performed immunohistochemical analysis of ADHFE1 protein expression in 243 CRC cohort groups. Immunohistochemistry was performed on formalin-fixed, paraffin-embedded tissue sections (4 μm thick). Sections were deparaffinized in xylene, rehydrated, and incubated with 0.3% hydrogen peroxide in methanol for 30 minutes. The sections were placed 10 mM Tris + 1 mM EDTA with a subsequent microwave antigen retrieval procedure. The sections were incubated with 4% BSA + Dextran/PBST to block nonspecific antibody binding, followed by incubation with the primary anti-ADHFE1 polyclonal antibody (Sigma-Aldrich, St. Louis, MO, USA) diluted 1:2500 in PBS with 0.1% Tween and 0.5% BSA. Sections were incubated with secondary antibody against HRP-conjugated-rabbit Ig, and bound antibody was visualized using 3, 3-diaminobenzidine substrate as a chromogen (Dako, Copenhagen, Denmark) followed by hematoxylin counter staining.

To assess ADHFE1 expression during mouse gut differentiation and development, six pregnant female mice (Orient Bio Inc., Seongnam, Korea). On 15, 17 and 18 days after implantation, mice were killed by CO_2_ inhalation, and each embryo was resected and frozen in liquid nitrogen for ADHFE1 immunohistochemistry. The immunohistochemistry method was the same as above. Mice were handled at the institute’s (Samsung Medical Center, Seoul, Korea) animal facility, and all treatments were in accordance with the legal and institutional guidelines.

### Immunoreactivity score (IS) in CRC tissues

In this study, we used the scoring method of Sinicrope *et al.* for evaluation of both the intensity of immunohistochemical staining and the proportion of stained epithelial cells [13]. The staining intensity was further classified as follows: weak, moderate, or strong. Positive cells were quantified as a percentage of the total number of epithelial cells and were assigned to one of the following five categories: 0, <5%; 1, 5%–25%; 2, 26%–50%; 3, 51%–75%; and 4, >75%. The percentage of positivity of epithelial cells and staining intensities were then multiplied to generate the IS for each case. For example, if the staining intensity was strong and the percentage of positive cells was greater than 75%, then the IS would be 3 × 4 = 12. As a result, the IS range was from 0 to 12. Each lesion was examined and scored by one pathologist (SHK). In the evaluation, membranous staining was considered positive.

### Cell culture and transfection

DLD-1 was cultured in IMDM (Invitrogen, Carlsbad, CA, USA) supplemented with 10% heat-inactivated fetal bovine serum (Invitrogen, Carlsbad, CA, USA). The full-length *ADHFE1* cDNA (Origene Technologies, Inc., Rockville, MD, USA) was subcloned into a pcDNA3.1(−) vector (Invitrogen, Carlsbad, CA, USA) to create pcDNA3.1(−)-*ADHFE1*. DLD-1 was transfected with pcDNA3.1(−)-*ADHFE1* or empty vector (pcDNA3.1(−)) by using Lipofectamine 2000 Reagent (Invitrogen, Carlsbad, CA, USA) according to the manufacturer’s protocol.

### Alkaline phosphatase (ALP) activity assay

ALP activity was determined according to the manufacturer’s protocol (Cell biolabs Inc., San Diego, CA, USA). DLD-1cells were seeded into 12 well plates at 3×10^5^cells/ml. After 72 hours, we gently aspirated the medium from the cells and washed the cells twice with cold PBS. Cells were lyzed in Cell Lysis Buffer (each 0.2 mL for 12 well dishes), incubated for 10 minutes at 4°C and removed from the solution and the cell debris spun down at 12,000 × g for 10 minutes. We performed a BCA assay to determine the protein concentration of the cell lysate. We added 50 μL of cell lysate to a 96-well plate. And then we prepared blank wells containing 50 μL Cell Lysis Buffer. We added 50 μL of StemTAG™ AP Activity Assay Substrate and incubated for 10–30 minutes at 37°C. After stopping the reaction by adding 50 μL of 1X Stop solution, we read the absorbance of each well at 405 nm.

### Statistics analysis

Statistics were calculated using SPSS software (SPSS v.20.0; IBM Corp., Armonk, NY, USA). Continuous data are presented as a mean ± standard deviation. Categorical data are presented as percentages. The comparison of IS among each group was evaluated using the Kruskal-Wallis H test because of the skewed distribution. For all tests, a *p* value < 0.05 was considered statistically significant. The difference in the continuous data of ALP activity assay among categorized groups was evaluated using Mann–Whitney *U* test because of a skewed distribution.

## Results

### Correlation between *ADHFE1* promoter methylation and ADHFE1 down-regulation in CRC cell lines

**Figure 1:**
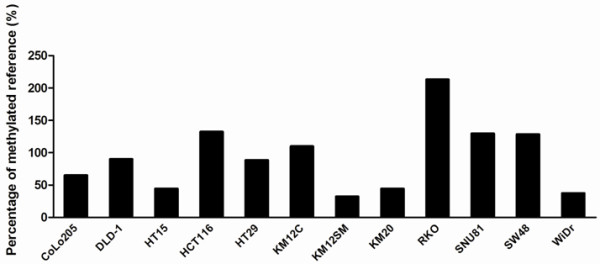
**The percentage of *ADHFE1 *methylated reference of CRC lines.** All CRC cell lines showed more than 10% PMR. The *ADHFE1* promoter was hypermethylated in all CRC cell lines.

**Figure 2:**
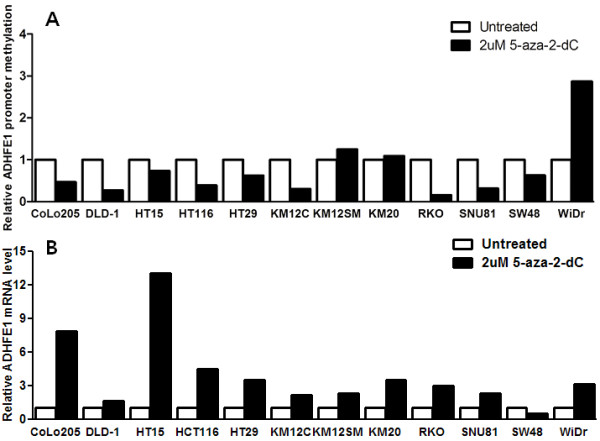
***ADHFE1 *promoter methylation and mRNA expression with and without treatment with 5-aza-2-dC.** (**A**) After treatment of 5-aza-2-dC, the relative methylation of *ADHFE1* promoter was decreased in 9 of 12 CRC cell lines. (**B**) After treatment of 5-aza-2-dC, the expression of *ADHFE1* mRNA was increased in 11 of 12 CRC cell lines.

To assay the aberrant DNA methylation of *ADHFE1*, we examined the 12 CRC cell lines by methylation-specific PCR. Despite being somewhat different, all 12 CRC cell lines showed more than 10% PMR (Figure 1). After the treatment of 5-aza-2-dC, we could observe the definite demethylating effect of 5-aza-2-dC in 9 of 12 CRC cell lines with the exception of KM12SM, KM20 and WiDr. Also, we measured the expression of *ADHFE1* mRNA in 12 CRC cell lines to investigate whether promoter methylation was associated with the inhibition of gene expression. After 5-aza-2-dC treatment, the expression of *ADHFE1* mRNA was up-regulated except SW48 (Figure 2).

### Correlation between *ADHFE1* promoter methylation and ADHFE1 down-regulation in CRC tissues

**Figure 3:**
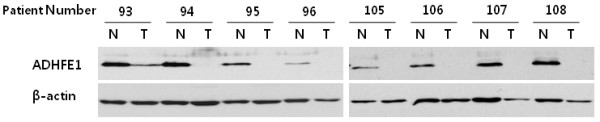
**The expression of ADHFE1 protein in CRC.** A western blotting analysis was performed to examine ADHFE1 protein expression in CRC (T) and adjacent normal colorectal tissue (N). These 8 blots are representative. The corresponding reprobings of these blots with actin as a loading control.

To examine the methylation status of the *ADHFE1* promoter in 124 paired CRC tissue and adjacent normal colorectal mucosa, and 59 independent advanced adenoma tissues, we performed methylation-specific PCR. The prevalence of *ADHFE1* promoter methylation in CRC and advanced adenoma was higher as compared with adjacent normal colorectal mucosa (Table 2). In addition, we examined whether methylation of the CpG Island in the *ADHFE1* promoter is associated with gene silencing by *ADHFE1* mRNA expression in the 27 paired CRC tissue and adjacent normal colorectal mucosa. Compared to that of the adjacent normal colorectal mucosa, the expression of *ADHFE1* mRNA was down-regulated in 96.3% (26/27) of CRC tissue. In addition, we analyzed the ADHFE1 protein expression in 36 paired CRC tissues and adjacent normal colorectal mucosa using a western blot. The expression of the ADHFE1 protein was down-regulated in 91.7% (33/36) of the CRC tissue. Therefore, it confirmed that the down-regulation of ADHFE1 in CRC tissues was significant compared to adjacent normal colorectal mucosa (Figure 3).

**Table 2:** ***ADHFE1 *hypermethylation and tumor type**

** Type of tissue**	**Number of tissue (%)**		***p *value**
	**Methylated**	**Unmethylated**	
CRC	101 (81.5)	23 (18.5)	<0.001
Advanced adenoma	50 (84.7)	9 (15.3)	
Normal colon	10 (8.1)	114 (91.9)	

CRC, colorectal cancer.

### Bisulfite genomic sequencing

**Figure 4:**
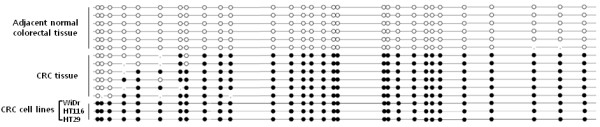
**The bisulfite genomic sequencing of the *ADHFE1 *promoter region.** Filled (black) circles correspond to the methylated CpG Island, unfilled (white) circles correspond to unmethylated CpG Island.

To confirm the methylation-specific PCR results in CRC cell lines and tissues, we selected 6 paired CRC and adjacent normal colorectal mucosa, and 3 CRC cell lines, such as WiDr, HT 116 and HT29, which had shown hypermethylation of *ADHFE1* promoter in methylation-specific PCR. As shown in Figure 4, a good association was seen between the methylation status, as assessed by methylation-specific PCR and the bisulfite sequence of the *ADHFE1* promoter.

### Localization of ADHFE1 protein determined by immunohistochemistry in CRC and normal colorectal mucosa

**Figure 5:**
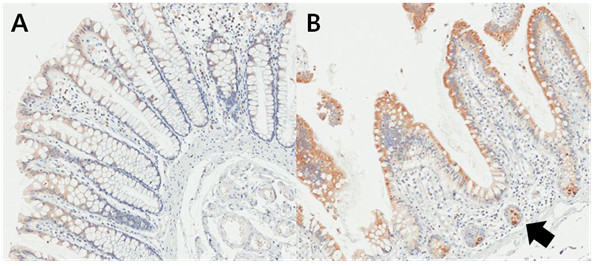
**Localization of ADHFE1 in normal colorectal mucosa (×200).** (**A**) The ADHFE1 expression was detected predominantly in the apex of the crypts. It was noted to be decreased toward the bottom of the crypts. (**B**) A strong expression of ADHFE1 can be observed in the Paneth cells (arrow).

ADHFE1 localization in the normal colorectal mucosa is confined to the surface epithelium and crypt top, and cells in the crypt base are unreactive. In other words, the intensity of ADHFE1 staining varied from the basal compartment to the top of the crypt epithelium. Epithelial cells at the crypt top showed a very strong ADHFE1 expression, whereas ADHFE1 staining was much weaker or none at the crypt base (Figure 5A). ADHFE1 staining was also seen on the proximal colon or terminal ileum in the regions where the presence of Paneth cells had a role in differentiation (Figure 5B). Given the differentiation gradient present from the basal compartment to the top of the crypt epithelium, ADHFE1 expression seemed modulated during differentiation.

**Figure 6:**
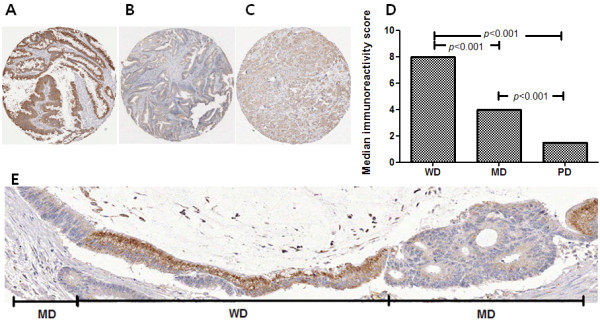
**The expression of ADHFE1 according to the differentiation status in CRC.** (**A**-**C**) Good differentiation is associated with the strong expression of ADHFE1. (**A**), well differentiated CRC; (**B**), moderate differentiated CRC; (**C**), poorly differentiated CRC (×5). (**D**) There is a correlation between ADHFE1 expression and differentiation status in CRC. (**E**) A representative case shows a simultaneously taken picture with a different gradient of differentiation in CRC (×100). MD, moderate differentiated CRC; WD, well differentiated CRC.

In CRC, ADHFE1 reactivity was seen throughout the crypt. These findings were different from those of normal mucosa. Samples were divided into three categories according to the differentiation. The good differentiation level was significantly associated with high ADHFE1 expression (Figure 6).

### Differentiation marker analysis after pcDNA3.1-*ADHFE1* transfection

**Figure 7:**
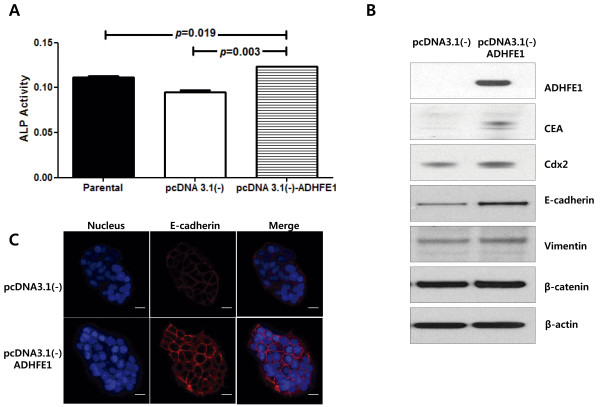
**The overexpression of ADHFE1 induces the expression of a colon differentiation marker.** (**A**) The ALP activity was increased in the DLD-1 cell line transfected with pcDNA3.1 vector containing the coding region of *ADHFE1*, compared to controls. (**B**) The expression of ADHFE1, CEA and Cdx2 is increased in the DLD-1 cell line transfected with pcDNA3.1 vector containing the coding region of *ADHFE1* and controls.

To investigate the function of ADHFE1, we selected a DLD-1 among the CRC cell lines, without expression of *ADHFE1*. We transfected the DLD-1 with pcDNA3.1 vector containing the coding region of *ADHFE1*. To substantiate this finding, the level of differentiation marker, including ALP, CEA and Cdx2, were determined. The activity of ALP was higher in pcDNA3.1-*ADFHE1* transfected cell colons compared with the empty vector transfectants and the parental cells with consistent correlation with ADHFE1 protein. The difference was statistically significant with a *p* value of < 0.05. Similarly, the expression of CEA was up-regulated in cell clones. In addition, the expression of Cdx2 had tended to be up-regulated in cell colons, but there was no significant difference of Cdx2 expression. These findings suggest that the expression of ADHFE1 induces CRC cell differentiation (Figure 7).

### ADHFE1 expression during mouse guts differentiation and development

**Figure 8:**
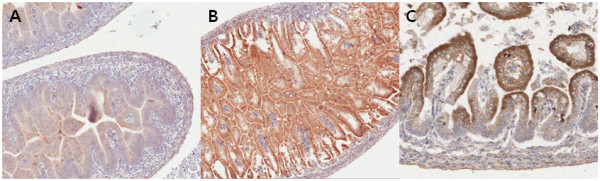
**The expression of ADHFE1 during mouse intestinal differentiation and development.** (**A**) A broad and diffuse staining of ADHFE1 is found in the early stage of gut at E15.5 (×5). (**B**) With progressive villous formation, ADHFE1 is expressed along the whole anterior-posterior axis of epithelium at E17.5 (×5). (**C**) At E18.5, ADHFE1 expression is more delineated in the top of the epithelium as differentiation proceeds (×20).

To gain insight into the role of ADHFE1 in embryo development and differentiation, we examined the ADHFE1 expression using immunohistochemistry at distinct developmental stages such as E15.5, E17.5 and E18.5. In mouse embryos, ADHFE1 expression of the large intestine was first detected at E15.5 (Figure 8A). This pattern was weak in the scattered cells of a developing gut. Additional expression began as the gut developed. At E17.5, the heaviest expression occurred with crypt formation (Figure 8B). At E18.5, ADHFE1 was predominantly expressed on the top of the mature crypt epithelium (Figure 8C). Collectively, these data suggest strongly that ADHFE1 may play a role in development and differentiation of embryo gut.

## Discussion

We report that the *ADHFE1* gene is frequently hypermethylated in CRC cell lines and tissues, using methylation-specific PCR and bisulfite genomic sequencing. *ADHFE1* mRNA levels are frequently down-regulated in CRC cell lines and tissues. When dense methylation of the *ADHFE1* promoter was restored after 5-aza-2-dC treatment, expression of *ADHFE1* mRNA and ADHFE1 protein had increased. Therefore we confirmed that *ADHFE1* methylation is under epigenetic regulation. In our study, some CRC cell lines such as KM12SM, KM20 and WiDr didn’t have the 5-aza-2-dC effect. Mossman D *et al.* found that 5-aza-2-dC induces gene expression, but is not necessarily dependent on DNA demethylation [14]. Therefore, all CRC lines didn’t observe the definite demethylating effect of 5-aza-2-dC in our study. In addition, the SW48 cell line had periods of rapid re-methylation and restoration with exposure of 5-aza-2-dC [14]. It is possible that the increased *ADHFE1* mRNA by 5-aza-2-dC in the SW 48 cell line was not detected.

According to the results of immunohistochemical staining, ADHFE1 expression is significantly reflected by the state of differentiation in CRC tissue. ADHFE1 expression in well differentiated CRC tissues was more intense than that in poorly differentiated CRC tissues, with statistical significance. We could confirm this finding from a representative case that simultaneously showed well and moderately differentiated CRC cells. In addition, ADHFE1 is expressed at the top of the colon with a decreasing gradient toward the crypt along the colon crypt axis in normal colon epithelium. This explains why ADHFE1 is also associated with the differentiation of normal colon epithelium. To obtain more information about the expression of ADHFE1 protein in embryo, we performed an immunohistochemical analysis according to respective mouse embryo steps. Our expression study showed that the ADHFE1 protein is highly related to mouse embryogenesis. Even though the data is not shown, ADHFE1 is expressed in the organs with active morphogenetic activities, such as the gut, lung, kidney and several glandular structures. In a mature embryo gut, ADHFE1 exhibits different expression patterns along the anterior-posterior axis of the gut. At E18.5, ADHFE1 expression is confined to the ventral part of gut. It is similar to ADHFE1 expression in the mature gut. Although immunohistochemical analysis is not a quantitative strategy, it was informative enough and a discernible method to show ADHFE1 expression in the epithelial cell layer of mouse embryo. Therefore, it seems that ADHFE1 may play a role in the differentiation and development of the developing gut in mouse embryo.

To confirm the association between ADHFE1 and differentiation, we choose DLD-1 CRC cell line without endogenous *ADHFE1* expression. The DLD-1, a typical poorly differentiated CRC cell line, has been known to have no endogenous CEA and ALP [15,16]. We confirmed the role of ADHFE1 for the increase of a well-known colon epithelium differentiation marker, such CEA and ALP [17]. Cdx2 is a caudal-related homeobox transcription factor. Unlike CEA and ALP, DLD-1 is well known to express Cdx2 [18]. Therefore, we might be able to find the definite difference of Cdx2 expression between the vector and *ADHFE1*-transfected cells [18]. Although it was not same focus, Kim *et al.* have shown a differentiation-dependent expression of ADHFE1 in adipogenesis [10].

## Conclusions

We first report that the hypermethylation of *ADHFE1* promoter in CRC is in concordance with the down-regulation of *ADHFE1* mRNA and protein. ADHFE1 protein has an important role in cell differentiation of normal colorectal mucosa and embryonic developmental processes. In addition, epigenetic inaction of *ADHFE1* modulates cell differentiation in CRC.

## Abbreviations

ADHFE1: Alcohol Dehydrogenase, iron containing, 1; ALP: Alkaline Phosphatase; CRC: Colorectal Cancer; IS: Immunoreactivity Score; PMR: Percentage of Methylated Reference; TSS: Translation Start Site.

## Competing interests

The authors declare that they have no competing interests.

## Authors’ contributions

CHT analysed the data and wrote the paper. KJY carried out experiments and analysed the data. SHK reviewed the hisopathological diagnosis of patients and provided the interpretation of the immunohistochemical staining results. SHK and HCK were involved in the collection of the study population. BHM, DKC, PLR and JCR critically read and evaluated the manuscript. JJK advised the protocol and analysed the data. YHK designed the study protocol, analysed the data and supervised the writing of the paper. All authors had final approval of the submitted and published versions.

## Pre-publication history

The pre-publication history for this paper can be accessed here:

http://www.biomedcentral.com/1471-2407/13/142/prepub
